# Glutamine prevents high-fat diet-induced hepatic lipid accumulation in mice by modulating lipolysis and oxidative stress

**DOI:** 10.1186/s12986-024-00784-1

**Published:** 2024-03-08

**Authors:** Yongjie Zhang, Yangli Wang, Xin Liao, Tong Liu, Fengyuan Yang, Kaiqiang Yang, Zhuohua Zhou, Yinxu Fu, Ting Fu, Aliaksei Sysa, Xiandan Chen, Yao Shen, Jianxin Lyu, Qiongya Zhao

**Affiliations:** 1 School of Basic Medical Sciences and Forensic Medicine, Hangzhou Medical College, Hangzhou, Zhejiang China; 2 Key Laboratory of Biomarkers and In Vitro Diagnosis Translation of Zhejiang Province, School of Laboratory Medicine and Bioengineering, Hangzhou Medical College, Hangzhou, China; 3 Key Laboratory of Laboratory Medicine, Ministry of Education, Zhejiang Provincial Key Laboratory of Medical Genetics, College of Laboratory Medicine and Life Sciences, Wenzhou Medical University, Wenzhou, China; 4 Zhejiang Provincial People’s Hospital, Affiliated People’s Hospital, Hangzhou Medical College, Hangzhou, China; 5 School of Public Health, Hangzhou Medical College, Hangzhou, China; 6 Belarusian State University, ISEI BSU, Minsk, Republic of Belarus

**Keywords:** Glutamine, Metabolic associated fatty liver disease, Prevention study, Reversal study, Oxidative stress

## Abstract

Metabolic-associated fatty liver disease (MAFLD) is related to metabolic dysfunction and is characterized by excess fat storage in the liver. Several studies have indicated that glutamine could be closely associated with lipid metabolism disturbances because of its important role in intermediary metabolism. However, the effect of glutamine supplementation on MAFLD progression remains unclear. Here, we used a high-fat diet (HFD)-induced MAFLD C57BL/6 mouse model, and glutamine was supplied in the drinking water at different time points for MAFLD prevention and reversal studies. A MAFLD prevention study was performed by feeding mice an HFD concomitant with 4% glutamine treatment for 24 weeks, whereas the MAFLD reversal study was performed based on 4% glutamine treatment for 13 weeks after feeding mice an HFD for 10 weeks. In the prevention study, glutamine treatment ameliorated serum lipid storage, hepatic lipid injury, and oxidative stress in HFD-induced obese mice, although glutamine supplementation did not affect body weight, glucose homeostasis, energy expenditure, and mitochondrial function. In the MAFLD reversal study, there were no noticeable changes in the basic physiological phenotype and hepatic lipid metabolism. In summary, glutamine might prevent, but not reverse, HFD-induced MAFLD in mice, suggesting that a cautious attitude is required regarding its use for MAFLD treatment.

**Supplementary Information:**

The online version contains supplementary material available at 10.1186/s12986-024-00784-1.

## Introduction

Fatty liver disease is a universal chronic liver disease that includes alcoholic and nonalcoholic fatty liver disease (NAFLD)(1, 2). As the understanding of the pathological features of NAFLD deepens, an expert group proposed the use of the term metabolic dysfunction-associated fatty liver disease (MAFLD) to replace NAFLD in 2020[3, 4]. This proposal aims to diminish the emphasis on alcohol as a defining factor in NAFLD and instead highlights the significance of metabolic risk factors that contribute to the progression of the NAFLD-associated pathology.

MAFLD has become a public health problem that threatens a quarter of the global population(5). Based on its histology, this disease includes simple hepatic steatosis, nonalcoholic fatty liver disease, and nonalcoholic steatohepatitis(6–8). MAFLD is associated with several metabolic diseases, including diabetes, obesity, and hypertension(9, 10). However, there is no clear explanation for its pathogenesis. Whereas the classic “multiple hit” theory partly explains that MAFLD might be associated with many metabolic factors, such as insulin resistance, obesity, and hormones, among others [11–13], there are still many gaps in our knowledge of the specific pathogenic mechanisms underlying MAFLD, and this requires further exploration.

As MAFLD is closely related to T2DM and obesity, most previous studies have focused on the roles of glucose and lipid metabolism in its pathogenesis(14, 15). More recently, with the development of metabolomics technology, amino acid dysmetabolism was found to have an essential role in liver lipid accumulation(16, 17). Some studies have also reported that circulating amino acids could serve as biomarkers of MAFLD(18–20). Further, based on the results of a cohort study, branched-chain amino acids (isoleucine, leucine, and valine) and aromatic amino acids (tryptophan, phenylalanine, and tyrosine) are more abundant, and glutamine, serine, and tyrosine levels are lower in patients with this disease(21). Thus, dietary amino acids could be an attractive method for preventing and reversing MAFLD.

Glutamine, a non-essential amino acid, is the most abundant amino acid in circulation. In addition to its role in protein synthesis, it is also involved in several metabolic pathways, including nucleotide and glutathione synthesis(22). In some metabolic diseases, the amount of glutamine produced in the body is insufficient to meet normal metabolic needs(23–26). Therefore, chronic glutamine deficiency can affect health. Epidemiological evidence suggests an association between lower circulating glutamine levels and MAFLD, implying that this amino acid regulates liver metabolism(27, 28). Studies have also shown that glutamine can regulate glucose metabolism by delaying gastric emptying [23]. Furthermore, glutamine decreases peroxide damage in adipose tissue by mitigating oxidative stress and inflammation(23, 29). However, it is unclear whether it has a protective effect against liver damage in high-fat diet (HFD)-fed mice. In this study, we performed disease prevention and reversal studies to evaluate the effect of glutamine on HFD-induced MAFLD.

## Results

### Glutamine-based treatment in the prevention study does not lower weight but improves serum lipid metabolism

**Fig. 1:**
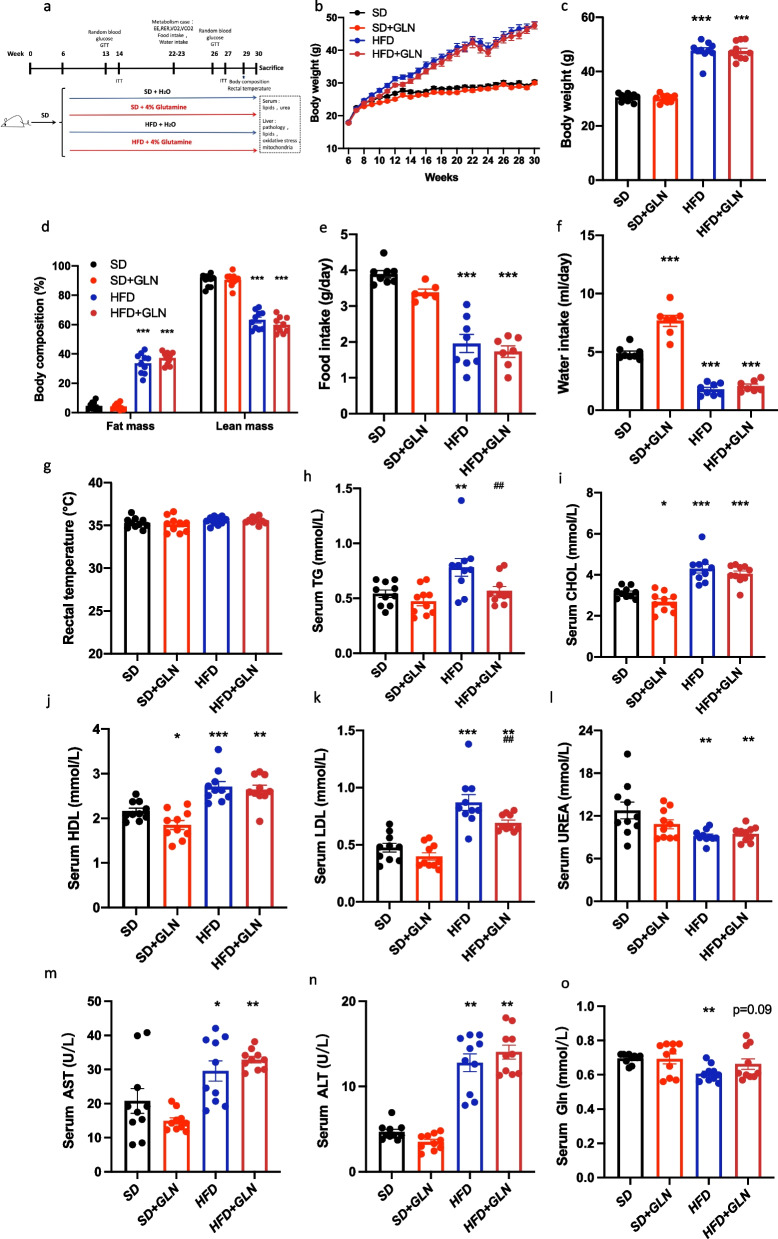
Glutamine-based treatment in the prevention study does not decrease weight gain but improves serum lipid metabolism in mice with diet-induced obesity. **a** Experimental design of the prevention study. **b** Curve of body weight changes (n = 10). **c** Body weights before euthanizing the mice (n = 10). **d** Body composition (n = 10). **e** Food intake (n = 6 to 8). **f** Water intake (n = 7 to 8). **g** Rectal temperature (n = 10); **h** Serum TG levels (n = 10). **i** Serum CHOL (n = 10). **j** Serum HDL (n = 10). **k** Serum LDL (n = 10). **l** Serum urea (n = 10). **m** Serum AST (n = 10). **n** Serum ALT (n = 10). **o** Serum Gln (n = 10). Values are shown as the mean ± SEM. * Significantly different from the SD group. # Significantly different from the HFD group. Significance: * or #, p < 0.05; ** or ##, p < 0.01; *** or ###, p < 0.001. TG, triglycerides; CHOL, cholesterol; HDL, high-density lipoprotein; LDL, low-density lipoprotein

To investigate the preventive effects of glutamine on MAFLD pathogenesis, we devised an experimental approach to model its progression in mice (Fig. 1a). After 24 weeks of standard diet (SD) or HFD feeding, body weight and fat mass were notably increased in the HFD-fed mice, as compared to those in SD-fed mice. However, glutamine treatment did not affect the body weight or body composition in either the SD or HFD groups (Fig. 1b–d). Food and water intake were slightly higher in the SD group than in the HFD group, but glutamine treatment did not affect food intake. Interestingly, glutamine increased water intake in the SD group (Fig. 1e, f). To understand whether glutamine treatment alters energy metabolism, we measured rectal temperatures and metabolic parameters. Rectal temperatures were equivalent among the four groups (Fig. 1g). Meanwhile, mice fed the SD showed significantly higher respiratory exchange ratio (RER), energy expenditure (EE), VO_2_, and VCO_2_ values than those fed an HFD. However, there was no difference in the EE, RER, VO_2_, and VCO_2_ based on glutamine treatment (Additional file 1: Figure S1a–d). To assess the effect of glutamine on HFD-induced alterations in serum metabolites, we analyzed the serum lipid profiles. As shown in Fig. 1h–n, triglyceride (TG), cholesterol (CHOL), low-density lipoprotein (LDL), high-density lipoprotein (HDL), aspartate aminotransferase (AST), and alanine aminotransferase (ALT) levels were significantly higher in HFD mice than in SD mice. In contrast, glutamine treatment resulted in lower serum TG and LDL contents, compared with those in untreated HFD-fed mice, and lower CHOL and HDL contents, compared with those in untreated SD-fed mice. Meanwhile, the serum urea content in the SD group was higher than that in the HFD group; however, glutamine treatment did not alter serum urea levels (Fig. 1l). The serum glutamine content in the SD group was higher than that in the HFD group, and glutamine treatment resulted in higher serum Gln contents compared with those in untreated HFD-fed mice.Although there was no significant difference between the glutamine treatment group and the untreated HFD-fed group, there was a tendency to increase serum glutamine content in the glutamine treatment group (Fig. 1o).

### Glutamine-based treatment in the prevention study does not improve glucose homeostasis

**Fig. 2:**
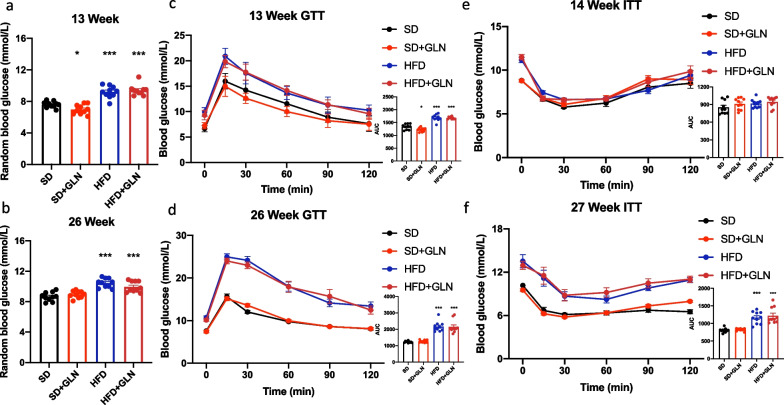
In the prevention study, glutamine-based treatment does not improve glucose homeostasis in mice with diet-induced obesity. **a** Random blood glucose 13 weeks after birth (n = 10). **b** Random blood glucose after 26 weeks of birth (n = 10). **c** GTT and the AUC of the GTT 13 weeks after birth (n = 10). **d** GTT and the AUC of the GTT 26 weeks after birth (n = 10). **e** ITT and AUC of the ITT 14 weeks after birth (n = 10). **f** ITT and the AUC of the ITT 27 weeks after birth (n = 10). Values are shown as the mean ± SEM. * Significantly different from the SD group. Significance: *, p < 0.05; **, p < 0.01; ***, p < 0.001. GTT, glucose-tolerance test; ITT, insulin-tolerance test; AUC, area under the curve

To evaluate the effect of glutamine on glucose homeostasis, we performed random blood glucose tests, glucose-tolerance tests (GTTs), and insulin-tolerance tests (ITTs) after both short- and long-term treatments. Mice fed an HFD had higher random blood glucose levels than those fed the SD. However, there was no apparent difference in random blood glucose levels after glutamine treatment (Fig. 2a, b). Moreover, HFD-fed mice had worse glucose tolerance and insulin sensitivity than SD mice. However, short- or long-term glutamine treatment had no significant effect on glucose homeostasis (Fig. 2c–f).

### Glutamine-based treatment in the prevention study has protective effects on HFD-induced liver injury

**Fig. 3:**
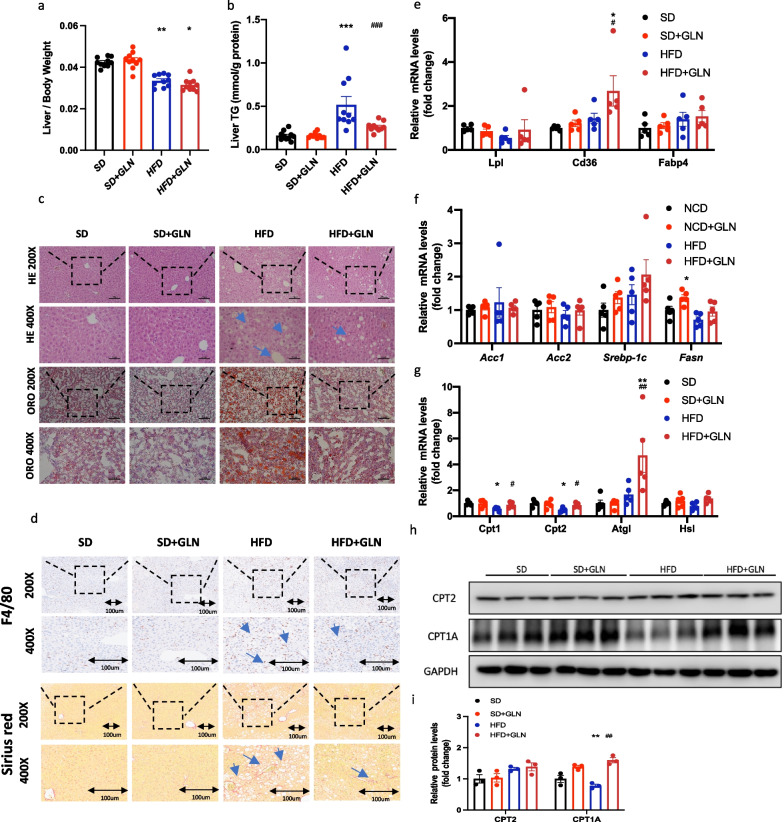
In the prevention study, glutamine-based treatment has protective effects on high-fat diet-induced liver injury. **a** Liver/body weight (n = 10). **b** Liver TG levels (n = 10). **c** Results of HE and ORO staining of liver sections. Blue arrows indicate hepatic steatosis. **d** Results of Sirius red and F4/80 staining of liver sections. **e** mRNA expression of lipid transport-related genes in liver tissue (n = 5). (**f**) mRNA expression of lipid synthesis-related genes (n = 5). **g** mRNA expression of lipolysis-related genes (n = 5). **h,i** Western blot analysis of proteins and densitometric quantification (n = 3).Values are shown as the mean ± SEM. * Significantly different from the SD group. # Significantly different from the HFD group. Significance: * or #, p < 0.05; ** or ##, p < 0.01; *** or ###, p < 0.001. TG, triglyceride; CHOL cholesterol; HE, hematoxylin and eosin; ORO, Oil Red O

To assess the influence of glutamine on MAFLD, we measured lipid accumulation in the liver tissue. After 23 weeks of feeding, the liver weight proportion in HFD-fed mice was decreased compared to that in SD-fed mice. However, glutamine treatment did not alter the liver weight ratio (Fig. 3a). Furthermore, liver TG levels were higher in HFD-fed mice than in SD-fed mice. Notably, glutamine treatment resulted in a reduction in liver TG levels in HFD-fed mice (Fig. 3b). Hematoxylin and eosin (H&E) staining of histopathological sections showed significant steatosis and the ballooning degeneration of hepatocytes in HFD-fed mice, which was improved by glutamine treatment (Fig. 3c). Oil Red O staining confirmed the decrease in lipid accumulation in the liver following glutamine-based treatment (Fig. 3c). At the same time, after glutamine treatment, the number of F4/80-positive cells and the positive Sirius red-red staining area were reduced, indicating that glutamine treatment can alleviate liver fibrosis and macrophage infiltration (Fig. 3d). To explore the mechanism underlying the reduction in lipid accumulation, we used quantitative real-time polymerase chain reaction (qRT-PCR) to measure the expression of genes involved in lipid metabolic processes in the liver, including lipid transport (*Lpl*, *Cd36*, and *Fabp4*), lipid synthesis (*Acc1*, *Acc2*, *Srebp-1c*, and *Fasn*), and lipolysis (*Cpt1*, *Cpt2*, *Atgl*, and *Hsl*). Gene expression related to synthesis was not significantly different among the four groups, but CD36 expression levels were elevated in the glutamine-treated group compared to those in HFD-fed mice, suggesting that glutamine helped reduce circulating lipid levels (Fig. 3e, f). However, remarkably, the expression levels of *Cpt1*, *Cpt2*, and *Atgl* were lower in the HFD group than in the SD group, indicating that an HFD might reduce lipolysis in the liver. In contrast, the reduction in lipolysis in HFD-fed mice was repressed by glutamine treatment (Fig. 3g). Compared with that in the SD group, the protein expression of CPT1A was decreased in the HFD group and increased after glutamine treatment (Fig. 3h, i). Thus, glutamine treatment enhanced lipid catabolism and reduced lipid accumulation in mice fed an HFD.

### Glutamine-based treatment in the prevention study ameliorates oxidative stress but does not affect mitochondrial quality control (MQC) systems during HFD-induced liver injury

**Fig. 4:**
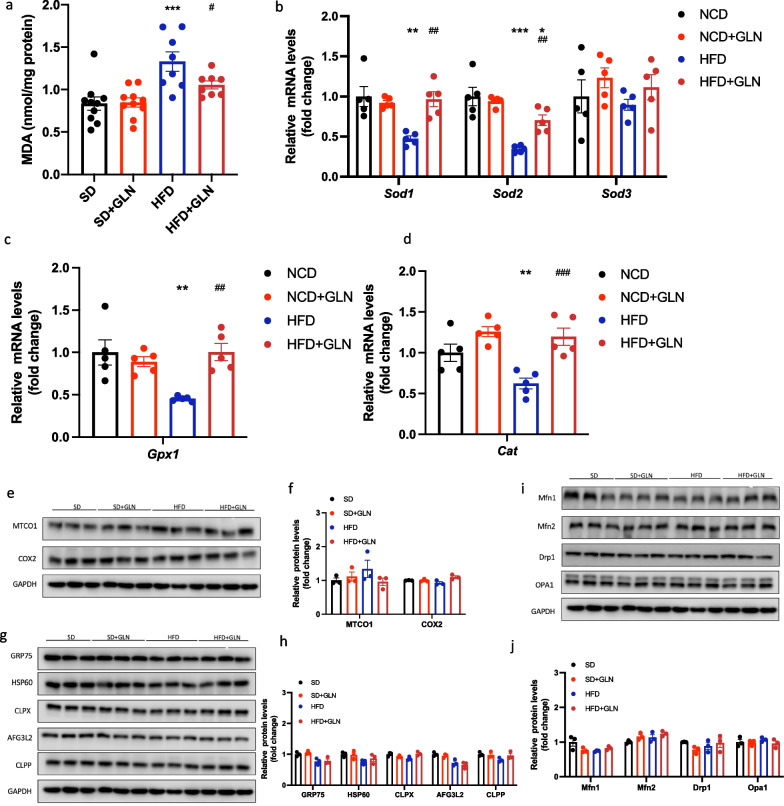
In the prevention study, glutamine-based treatment ameliorates oxidative stress but does not affect mitochondrial quality control (MQC) systems in response to high-fat diet-induced liver injury. **a** Liver MDA levels (n = 10). **b** mRNA expression of *SOD* (n = 5). **c** mRNA expression of *Gpx1* (n = 5). **d** mRNA expression of *Cat* (n = 5). **e**,** f** Western blot analysis of proteins encoded by mitochondrial genes and densitometric quantification (n = 3). **g**,** h** Western blot analysis of the molecular MQC systems and densitometric quantification (n = 3). **i**, **j** Western blot analysis of the MQC system components at the organelle level and densitometric quantification (n = 3). Values are shown as the mean ± SEM. * Significantly different from the SD group. # Significantly different from the HFD group. Significance: * or #, p < 0.05; ** or ##, p < 0.01; *** or ###, p < 0.001

To further understand the protective effects of glutamine treatment on the liver, we examined liver oxidative stress and MQC systems. Hepatic MDA levels, a marker of lipid peroxidation, were significantly elevated in mice fed the HFD. However, treatment with glutamine suppressed this effect (Fig. 4a). Furthermore, we measured the mRNA expression of superoxide dismutase (*SOD*). Here, a pronounced reduction in *SOD1* (encoding Cu–Zn SOD) and *SOD2* (encoding mitochondrial SOD) expression was detected in HFD-fed mice compared with that in the SD group; however, no change in SOD3 (extracellular SOD) expression was observed. Meanwhile, the mRNA levels of *SOD1* and *SOD2* were higher in the glutamine-treated group than in the untreated HFD-fed group (Fig. 4b). Moreover, the reduction in the mRNA expression of *Gpx1* and *Cat* mediated by the HFD was similarly suppressed by glutamine treatment (Fig. 4c, d). Some studies have shown that an HFD impairs mitochondrial functions. Thus, to investigate whether glutamine can have a protective role in this, we measured the expression of proteins involved in the MQC system. The protein expression of MTCOI and COX2, encoded by mitochondrial genes, was not significantly different among the four groups (Fig. 4e, f). We further analyzed the expression of proteins of the MQC systems at the molecular and organelle levels. Similarly, no changes were observed among the four groups (Fig. 4g–j).

### Glutamine-based treatment in the reversal study does not improve weight gain, glucose homeostasis, and serum lipid metabolism

**Fig. 5:**
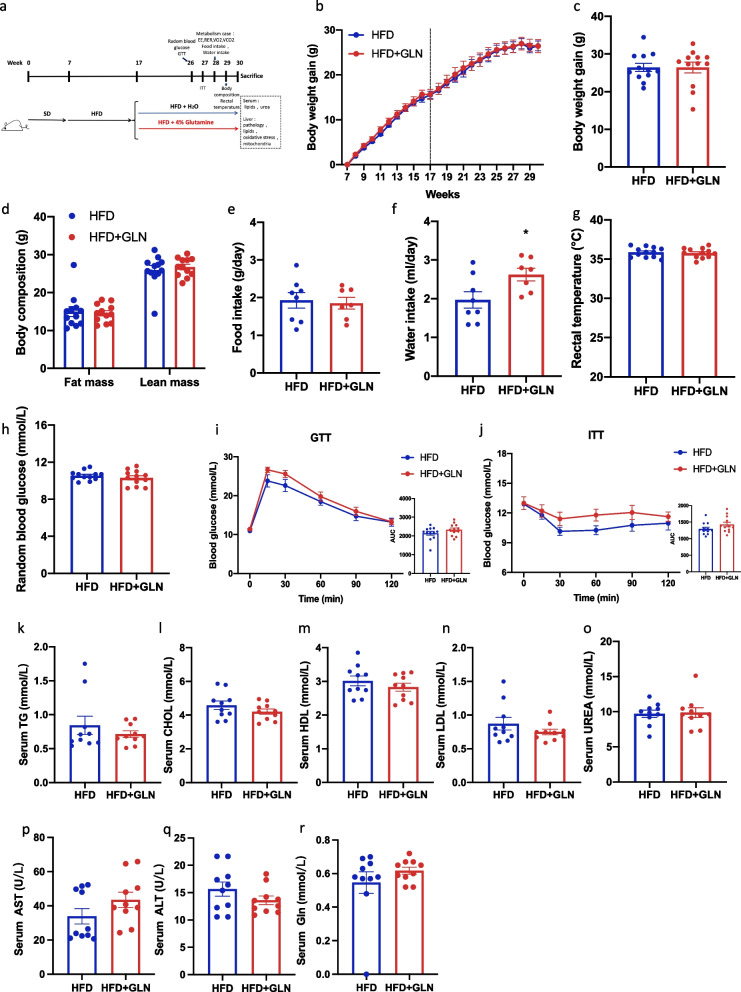
Glutamine-based treatment in the reversal study does not improve weight gain, glucose homeostasis, and serum lipid metabolism in mice with diet-induced obesity. **a** Experimental design of the reversal study. **b** Curve of body weight changes (n = 12). **c** Body weights before euthanizing the mice (n = 12). **d** Body composition (n = 12). **e** Food intake (n = 7–8). **f** Water intake (n = 7–8). **g** Rectal temperature (n = 12). **h** Random blood glucose (n = 12). **i** GTT (n = 12). **j** ITT (n = 12). **k** Serum TG levels (n = 10). **l** Serum CHOL (n = 10). **m** Serum HDL (n = 10). **n** Serum LDL (n = 10). **o** Serum urea (n = 10). **p** Serum AST (n = 10). **q** Serum ALT (n = 10). **r** Serum Gln (n = 10). Values are shown as the mean ± SEM. * Significant difference compared to the HFD group. Significance: *, p < 0.05; **, p < 0.01; ***, p < 0.001. GTT, glucose-tolerance test; ITT, insulin-tolerance test; TG, triglyceride; CHOL, cholesterol; HDL, high-density lipoprotein; LDL, low-density lipoprotein; AST, aspartate aminotransferase; ALT, alanine aminotransferase

To determine whether glutamine can be used to treat liver lipid accumulation, we devised a reversal experiment in which glutamine was administered after 10 weeks of HFD administration (Fig. 5a). After 13 weeks of glutamine treatment, there were no significant differences in body weight and composition compared to those in the untreated HFD group (Fig. 5b–d). Moreover, the average daily food intake did not change with glutamine treatment (Fig. 5e). However, the daily water intake was slightly increased after glutamine treatment (Fig. 5f). Similarly, there were no apparent alterations in the rectal temperature (Fig. 5g) or energy metabolism (EE, RER, VO_2_, and VCO_2_) (Figure S2a–d). These results suggest that glutamine does not regulate body weight through food intake or energy metabolism. To obtain further insights into the metabolism of glucose and lipids in this reversal study, we performed a GTT, ITT, and serum biochemistry test. At week 24, the random blood glucose level did not change after glutamine-based treatment (Fig. 5h). Similarly, we did not observe improved glucose tolerance or insulin sensitivity after the glutamine-based treatment (Fig. 5i, j). In addition, as shown in Fig. 5k–r, serum TG, CHOL, HDL, LDL, urea, AST, ALT, and Gln levels were not significantly different between the groups in the reversal study. Thus, glutamine-based treatments alone cannot reverse serum lipid dysregulation.

### Glutamine-based treatment in the reversal study does not improve HFD-induced liver injury

**Fig. 6:**
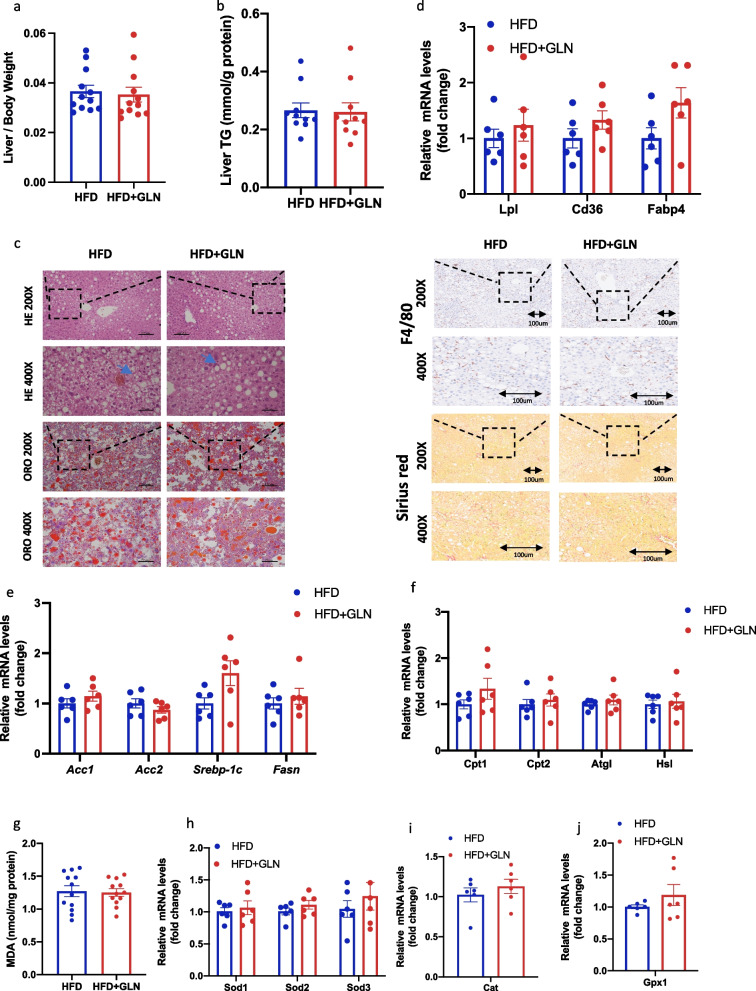
Glutamine-based treatment in the reversal study does not improve high-fat diet-induced liver injury. **a** Liver/body weight (n = 12). **b** Liver TG levels (n = 10). **c** Results of HE and ORO staining of liver sections. Blue arrows indicate hepatic steatosis. Results of Sirius red and F4/80 staining of liver sections. **d** mRNA expression of lipid transport-related genes in liver tissue (n = 6). **e** mRNA expression of lipid synthesis-related genes (n = 6). **f** mRNA expression of lipolysis-related genes (n = 6). **g** Liver MDA levels (n = 12). **h** mRNA expression of *SOD* (n = 6). **i** mRNA expression of *Gpx1* (n = 6). **j** mRNA expression of *Cat* (n = 6).Values are shown as the mean ± SEM. * Significant difference compared to the HFD group. Significance: *, p < 0.05; **, p < 0.01; ***, p < 0.001. TG, triglyceride; HE, hematoxylin and eosin; ORO, Oil Red O

In the prevention study, we observed that glutamine treatment could ameliorate HFD-induced liver injury. Therefore, we investigated the effect of glutamine on the liver tissue. In the reversal study, the liver weight ratio was not different between the groups (Fig. 6a). We then analyzed lipid accumulation in the liver. However, we did not find significant differences in liver TG levels upon glutamine treatment compared to those in the untreated HFD group (Fig. 6b). Next, we performed H&E, Oil Red O, Sirius red, and F4/80 staining of the liver sections. An analysis of pathological sections of the liver demonstrated that glutamine treatment did not reverse lipid accumulation, steatosis, ballooning degeneration, fibrosis, or macrophage infiltration in hepatocytes (Fig. 6c). Similarly, we examined lipid metabolism in the liver, including lipid transport, synthesis, and lipolysis. The qRT-PCR results indicated that glutamine treatment did not alter the expression of lipid metabolism-related genes (Fig. 6d–f). According to our previous study, glutamine exerts a protective effect against oxidative stress. Therefore, we assessed oxidative stress-related indicators in the liver. An analysis of the MDA content indicated that the glutamine-based treatment did not improve oxidative stress in the liver (Fig. 6g). Moreover, the mRNA expression levels of genes encoding key enzymes involved in antioxidative stress did not differ between groups in the reversal study (Fig. 6h–j). Together, these results indicated that glutamine treatment did not reverse HFD-induced liver injury.

## Discussion

Accumulating evidence indicates that amino acids play important roles in the development and progression of MAFLD(30). Population cohort studies have demonstrated that low circulating glutamine levels are closely associated with the risk of MAFLD. In some metabolic diseases, the amount of glutamine produced in the body is not sufficient to meet the normal metabolic needs. Moreover, healthy people can obtain glutamine through food and production in the body, whereas patients with fatty liver exhibit reduced synthesis in the body, and thus, the level of glutamine in the plasma is reduced(27). This suggests that glutamine supplementation is a potential dietary intervention that could prevent and reverse MAFLD. In this study on MAFLD prevention, we demonstrated that glutamine helps mitigate the severity of liver lipid damage in mice exposed to an HFD by ameliorating changes in serum lipids, liver lipid metabolism, and oxidative stress. These results indicate that glutamine can have a beneficial effect on the pathogenesis of MAFLD. It has been previously shown that oral glutamine has a protective effect on the development of HFD-induced NASH in mice, which is associated with the protection of the liver from the induction of iNOS and lipid peroxidation(31–33). Unlike in previous studies, we wondered whether glutamine could prevent or reverse the development of MAFLD. However, in our reversal study, we did not observe any obvious effects of glutamine on MAFLD. In general, we found that glutamine-based treatment has a protective role only (in prevention studies) by improving lipid metabolism and oxidative stress but does not reverse HFD-induced liver injury.

As expected, following HFD administration to C57BL/6J mice, we identified metabolic disorders, such as insulin resistance, lipid accumulation, and serum lipid abnormalities. Previous studies have suggested that glutamine has beneficial effects on preventing obesity in obese rats(34). However, we did not find that glutamine could reduce body weight gain in either the prevention or reversal studies after HFD administration. Our results are in accordance with those of Patricia et al. but differ from those of Kehinde et al.[35, 36]. We believe that this difference might be associated with the animal strain and the species used for HFD administration. Balancing energy intake and expenditure is vital for maintaining body weight(37, 38). To determine the role of glutamine in weight gain, we measured food intake and energy metabolic parameters using a metabolism cage. Food intake in the HFD group was lower than that in the SD group. As an HFD has a high energy density, long-term excessive energy intake leads to obesity(39). Meanwhile, HFD-fed mice had lower energy expenditure and RERs. Thus, weight gain associated with an HFD results from excessive energy intake and low energy expenditure. However, our results indicate that long-term glutamine supplementation did not alter the energy intake, EE, or RER in either the SD or HFD groups. In line with our results, in a previous study, the injection of glutamine into mice did not affect the food intake, EE, or the RER upon SD and HFD administration. These results further support the hypothesis that long-term dietary glutamine does not improve body weight changes in HFD-induced obese mice.

Further, in our study, only reduced random blood glucose levels were observed in the SD group at week 13. We thus believe that glutamine treatment minimally affected glucose metabolism. This is consistent with a study by Patricia et al., in which 8 weeks of L-glutamine supplementation did not improve glucose tolerance or insulin sensitivity after 20 weeks of HFD administration(35). In previous studies, it was speculated that the effect of glutamine on glycemic control in patients with diabetes could be due to delayed gastric emptying(40, 41). However, our results did not confirm that glutamine reduces food intake. Thus, we believe that glutamine has only a transitory effect on blood glucose control. During the GTT and ITT, the glutamine solution was replaced with water without glutamine in advance. Therefore, glutamine did not improve glucose homeostasis. Other studies have used a rat model treated with an injection of streptozotocin to study the glucose-lowering effects of glutamine(41–44). These factors could also explain why our results contradict those of other studies.

Lipid accumulation is an essential feature of MAFLD induced by an HFD. As expected, we found that mice fed an HFD had higher serum and liver TG levels than mice fed a SD. In a previous study, we found that glutamine treatment improves lipid accumulation. A pathological examination further revealed reduced liver damage and lipid accumulation following glutamine treatment. In addition, results based on mice with fructose-induced obesity showed that glutamine protects pregnant rats against hepatic lipid accumulation(36). However, this protective effect was not observed in our study. Thus, we speculate that glutamine can delay lipid accumulation. An assessment of the expression of lipid-related genes revealed that glutamine could ameliorate the decline in lipolysis. These results indicated that glutamine might protect against lipid accumulation by regulating lipid decomposition.

Excessive lipid accumulation in the liver results in oxidative stress and mitochondrial function disorders(45). Moreover, oxidative stress is a key contributor to the pathogenesis of MAFLD(46, 47). Glutamine is an important substrate for the de novo synthesis of glutathione, which is a potent antioxidant that protects cells against lipid peroxidation. Some studies have shown that glutamine supplementation increases the activity of antioxidant enzymes, including SOD, Gpx, and catalase(48, 49). Our analysis indicated that the lipid peroxidation product MDA was increased in the HFD group. In a previous study, glutamine treatment was shown to effectively reduce lipid peroxide damage. Numerous studies have also reported that MAFLD progression is associated with mitochondrial dysfunction(50, 51). Therefore, we examined the expression of MQC proteins. The MQC system changed only minimally during the progression of MAFLD. Our results are consistent with those of a previous study showing that HFD-induced MAFLD is not closely related to impaired mitochondrial function in the liver [52].

Our study had some limitations that could affect our understanding of the effect of glutamine in HFD-fed mice. As the solubility of glutamine in water is limited, we were unable to provide further evidence to validate the role of high glutamine concentrations. Some studies have indicated that food intake is a determining factor of MAFLD progression. Therefore, changes in dietary habits could reverse the adverse effects of MAFLD(31, 53). However, in this reversal study, we did not include a group based on dietary interventions to determine the effects of glutamine; accordingly, glutamine therapy has some potential but might need to be combined with other treatments. In summary, our study provides evidence that glutamine can improve lipid accumulation and reduce oxidative stress. However, glutamine might prevent, but not reverse, HFD-induced MAFLD in mice, suggesting that a cautious attitude is required regarding its use for MAFLD treatment.

## Materials and methods

### Animals

All animal experiments were approved by the Ethics Committee of the Wenzhou Medical University. All experimental protocols were performed in accordance with the code of the Animal Care and Use Committee of Wenzhou Medical University and complied with the Animal Research: Reporting In Vivo Experiments (ARRIVE) guidelines. Six-week-old male C57BL/6J mice were purchased from the Zhejiang Academy of Medical Sciences (Hangzhou, China) by the Animal Experimental Center of Wenzhou Medical University. All animals were healthy and housed under standard conditions (12 h/12 h light/dark cycle, 22 ± 2 °C), with free access to pelleted feed and autoclaved drinking water. Mice were allowed to acclimate to the environment for 1 week before experimentation. The mice were fasted 12 h in advance and anesthetized before treatment, and blood was taken via the eyeball, and then, tissue was dissected. The total number of mice in both the prevention and reversal experiments was 12. Four mice were randomly selected for the WB experiment, and 50% of the total number of samples (5–6 mice) were randomly selected for tissue sectioning, serology, and PCR analysis.

### Prevention study

Forty male mice were randomly divided into four groups as follows: SD + water, SD + water with 4% glutamine (V900419, Sigma-Aldrich, St. Louis, MO, USA) (SD + GLN), 60% HFD (Research Diet, New Brunswick, NJ, USA) + water (HFD), and HFD + water with 4% glutamine (HFD + GLN). A GTT was performed at 13 and 26 weeks, and an ITT was performed at 14 and 27 weeks. At 22–23 weeks, mice were placed in metabolic cages. During the last week of feeding, their body composition was measured using nuclear magnetic resonance (NMR), and their rectal temperature was determined using a rectal probe linked to a digital thermometer. The body weights of all mice were monitored weekly at the same time points.

### Reversal study

Twenty-four male mice were randomly assigned to two groups after 10 weeks of HFD administration. One group was fed an HFD with normal water, and the other group was fed an HFD containing a 4% glutamine solution (HFD + GLN). At 26 weeks, the mice were subjected to a GTT and random blood glucose test. After 1 week, mice were subjected to an ITT. At 28 weeks of age, the mice were housed in metabolic cages. During the last week of feeding, their body composition was measured using NMR, and their rectal temperature was determined using a rectal probe linked to a digital thermometer. The body weights of all mice were monitored weekly at the same time points.

### GTT

All drinking water was replaced with water without glutamine. After a 12 h period of fasting, the mice were injected intraperitoneally with glucose (1.5 g/kg body weight). At 0, 15, 30, 60, 90, and 120 min after injection, blood was taken from the tail vein, and blood glucose levels were assessed using a glucometer.

### ITT

All drinking water was replaced with water without glutamine. After a 6 h period of fasting, the mice were injected intraperitoneally with regular human insulin (0.75 U/kg body weight). At 0, 15, 30, 60, 90, and 120 min after injection, blood was taken from the tail vein, and blood glucose levels were assessed using a glucometer.

### Measurement of metabolic parameters

The mice were randomly selected and housed in metabolic cages for 1 week. All mice were permitted to adapt to the metabolic cage for 2 d before a 3 d consecutive measurement period. Oxygen consumption, carbon dioxide production, the RER, and heat production were measured via indirect calorimetry using TSE PhenoMaster metabolic cages (Bad Homburg, Germany) with sensors that sampled air from each case once every 30 min to collect data on respiratory functions and food and water intake.

### Body composition analysis

The body compositions of the mice were measured using quantitative magnetic resonance technology (Bruker), which can distinguish the differential proton states among fat mass, lean mass, and free water.

### Serum biochemistry

Serum CHOL, TG, HDL, LDL, urea (OSR60118, OSR6116, OSR6187, OSR6183, OSR6234, Beckman Coulter Inc., Georgia, USA), aspartate aminotransferase (AST), and alanine aminotransferase (ALT) (C010-2–1, C009-2–1, Nanjing Jiancheng Bioengineering Institute, China) were measured using commercial enzymatic kits on a Beckman AU480 chemistry analyzer (Beckman Coulter Inc., Atlanta, GA, USA).

### Hepatic TG and MDA contents

Liver samples were homogenized in lysis buffer (w/v = 10; 0.01 mol/L Tris–HCl, 0.0001 mol/L EDTA-2Na, 0.01 mol/L sucrose, and 0.8% NaCl solution [pH 7.4]). The lysates were centrifuged, and the supernatants were collected for the measurement of hepatic TG and MDA levels, which were measured using TG (H203-1–1/A1101-1; Nanjing Jiancheng, China) and MDA assay kits (A003-1–2; Nanjing Jiancheng, China) in accordance with the manufacturer’s instructions.

### Histopathology and immunohistochemistry staining

The liver tissues were rinsed in ice-cold phosphate-buffered saline and fixed with 4% paraformaldehyde at 4 °C for 24 h. The liver samples were then dehydrated in alcohol solutions of increasing concentrations from 30 to 100% and embedded in paraffin. The tissues were cut to a 5 µm thickness and then stained with H&E (C0105-1 and C0105-2, respectively; Beyotime, Shanghai, China) and Sirius red staining (G1472; Solarbio, Beijing, China). For Oil Red O (A600395-0050; Sangon Biotech, Shanghai, China) staining, paraformaldehyde-fixed tissues were dehydrated in a 30% sucrose solution for 48 h, embedded in OCT compound, and snap-frozen. Afterward, the samples were cut into 10 µm-thick sections using a Cryostat Microm HM 525 (Thermo Fisher Scientific, USA). For immunohistochemistry staining, tissue sections were incubated with primary antibodies against F4/80 (Abcam) at 4 °C overnight, followed by incubation with appropriate HRP-labeled secondary antibodies. The streptavidin–biotin detection system was applied, and 3, 3′-diaminobenzidine (DAB) was used. A light microscope (Nikon, Japan) was used to examine each histological segment.

### qRT-PCR

TRIzol reagent (Thermo Fisher Scientific) was used to extract total RNA from liver tissues. Then, 500 ng of total RNA was used for reverse transcription in a 10 µL reaction volume using 5 × Hiscript qRTSuperMIX (Perfect Real Time; Takara Biotechnology, China). Quantitative PCR was performed using SYBR Green Supermix and a Quantagene q225 (Kubo Tech, Beijing, China). The expression level of each target gene was normalized to that of the reference gene *Actin* and was calculated as 2^−∆CT^.

### Western blotting

Total proteins from liver tissue were extracted using RIPA lysis buffer (Cell Signaling Technology, USA) supplemented with the protease inhibitor phenylmethylsulfonyl fluoride (1 mM; Sangon Biotech, Shanghai, China) and boiled for 5 min at 95 °C. Proteins separated using BN-PAGE or SDS-PAGE were transferred to 0.22 mm polyvinylidene fluoride membranes (Bio-Rad, Hercules, CA) using a semidry transfer system (Bio-Rad) and probed with the following primary antibodies: anti-MTCO1 (ab14705; 1:1,000), anti-COX2 (376,731; 1:2,000), anti-mfn1 (ab126575; 1:1,000), anti-mfn2 (ab260861; 1:1,000); anti-DRP1 (ab184247; 1:1,000), anti-OPA1 (ab157457; 1:1,000), anti-HSP60 (ab190828; 1:1,000), anti-CLPX (ab168338; 1:1,000), anti-GRP75 (sc133137; 1:1,000), and anti-CLPP (ab124822; 1:1,000). Membranes were incubated with primary and secondary antibodies. The expression of each protein was compared with that of GAPDH (sc-365062; 1:2000).

### Statistical tests

All results are expressed as the mean ± standard error of the mean (SEM). All statistical analyses were performed using GraphPad Prism software (version 8.0; San Diego, CA, USA). All data conformed with a normal distribution. Multiple comparison analysis was performed using two-way ANOVA in the prevention study. Significant differences were analyzed by performing Student’s t-tests in the reversal study. The threshold for statistical significance was established at 0.05.

### Supplementary Information

**Additional file 1.** In the prevention and reversal study, glutamine-based treatment does not affect the energy balance in mice with diet-induced obesity.

## Data Availability

The data presented in this study are available on request from the corresponding author.
