# Supplementary material for: Glutamine prevents high-fat diet-induced hepatic lipid accumulation in mice by modulating lipolysis and oxidative stress
Source: Nutr Metab (Lond). 2024 Mar 8;21:12. doi: 10.1186/s12986-024-00784-1 (PMC10924388; doi:10.1186/s12986-024-00784-1)
Supplement: Supplementary file 1 — Additional file 1. In the prevention and reversal study, glutamine-based treatment does not affect the energy balance in mice with diet-induced obesity. [file 12986_2024_784_MOESM1_ESM.docx]

**Figure S1. Glutamine-based treatment in the prevention study does not affect the energy balance in mice with diet-induced obesity.** (**a**) RER and the average RER (n=8); (**b**) EE and average EE (n=8); (**c**). VO_2_ and average VO_2_ (n=8); (**d**) VCO_2_ and average VCO_2_ (n=8). Values are shown as the mean ± SEM. * Significantly different from the SD group. Signiﬁcance *, p < 0.05, **, p < 0.01, ***, p < 0.001. RER, respiratory exchange ratio; EE, energy expenditure.

**Figure S2. Glutamine-based treatment in the reversal study does not affect the energy balance in mice with diet-induced obesity.** (**a**) RER and average RER (n=8); (**b**) EE and average EE (n=8); (**c**) VO_2_ and average VO_2_ (n=8); (**d**) VCO_2_ and average VCO_2_ (n=8). Values are shown as the mean ± SEM. * Significantly different from the SD group. Signiﬁcance *, p < 0.05, **, p < 0.01, ***, p < 0.001. RER, respiratory exchange ratio; EE, energy expenditure.
